# A genome-wide association analysis for salt tolerance during the soybean germination stage and development of KASP markers

**DOI:** 10.3389/fpls.2024.1352465

**Published:** 2024-02-07

**Authors:** Junyan Wang, Miaomiao Zhou, Hongmei Zhang, Xiaoqing Liu, Wei Zhang, Qiong Wang, Qianru Jia, Donghe Xu, Huatao Chen, Chengfu Su

**Affiliations:** ^1^ College of Agronomy, Qingdao Agricultural University, Qingdao, China; ^2^ Institute of Industrial Crops, Jiangsu Academy of Agricultural Sciences, Nanjing, China; ^3^ Japan International Research Center for Agricultural Sciences (JIRCAS), Tsukuba, Ibaraki, Japan; ^4^ Zhongshan Biological Breeding Laboratory (ZSBBL), Nanjing, China

**Keywords:** soybean, salt tolerance, germination stage, genome-wide association analysis, KASP marker

## Abstract

Salt stress poses a significant challenge to crop productivity, and understanding the genetic basis of salt tolerance is paramount for breeding resilient soybean varieties. In this study, a soybean natural population was evaluated for salt tolerance during the germination stage, focusing on key germination traits, including germination rate (GR), germination energy (GE), and germination index (GI). It was seen that under salt stress, obvious inhibitions were found on these traits, with GR, GE, and GI diminishing by 32% to 54% when compared to normal conditions. These traits displayed a coefficient of variation (31.81% to 50.6%) and a substantial generalized heritability (63.87% to 86.48%). Through GWAS, a total of 1841 significant single-nucleotide polymorphisms (SNPs) were identified to be associated with these traits, distributed across chromosome 2, 5, 6, and 20. Leveraging these significant association loci, 12 candidate genes were identified to be associated with essential functions in coordinating cellular responses, regulating osmotic stress, mitigating oxidative stress, clearing reactive oxygen species (ROS), and facilitating heavy metal ion transport - all of which are pivotal for plant development and stress tolerance. To validate the candidate genes, quantitative real-time polymerase chain reaction (qRT-PCR) analysis was conducted, revealing three highly expressed genes *(Glyma.02G067700*, *Glyma.02G068900*, and *Glyma.02G070000)* that play pivotal roles in plant growth, development, and osmoregulation. In addition, based on these SNPs related with salt tolerance, KASP (Kompetitive Allele-Specific PCR)markers were successfully designed to genotype soybean accessions. These findings provide insight into the genetic base of soybean salt tolerance and candidate genes for enhancing soybean breeding programs in this study.

## Introduction

Soil salinization constitutes a pressing global concern, posing a significant threat to crop growth and food production (Zhai et al., 2023). Recent statistics reveal that approximately 23% of cultivated land worldwide is affected by soil salinization, with 1.1 billion hectares of global land area afflicted by this issue. China is not immune to this challenge, with a saline soil area encompassing 36.9 million hectares, a substantial 10% of the global saline soil extent, and accounting for 5% of China’s total available land (Zhao et al., 2022). China’s saline soil total area is 36.9 million hm^2^, accounting for 10% of the global saline soil, accounting for 5% of the country’s available land area (Zhao et al., 2022). This issue manifests diversely across regions, including coastal saline soil and sea mud along the eastern coast, salt-affected soil in the Huang-Huai-Hai Plain, saline soil in the northeast plain, salt-impacted soil in the northwest inland areas, and desert salt soil in Qinghai and Xinjiang (Mao et al., 2020). In response to this critical concern, ensuring food security has prompted state initiatives aimed at optimizing available land resources through systematic planning of saline-alkali land, the selection of salt-tolerant crops for soil amelioration, and the preservation of cultivated land areas (Luan et al., 2023).

Soybean, a member of the legume family and the Papilionoideae stands as a pivotal cash crop, oilseed, and edible plant protein source in China. It also plays a crucial role as an industrial raw material (Li, 2011; Meng et al., 2021; Lu et al., 2023; Wang et al., 2023). Moreover, soybean holds a unique distinction as the cornerstone of Sino-U.S. agricultural trade relations, drawing significant attention from researchers. Because of saline land on soybean yield of serious damage to make our country’s soybean production. Therefore, we cultivate salt-tolerant high yield soybean varieties of this work is particularly important.

Up to now, 1536 QTLs associated with salt tolerance have been reported, generally located on chromosomes 2, 3, 6, 8, 9, 12, 13, 14, and 17.(SoyBase.org) Two different materials were used to locate the QTL of soybean salt tolerance, and one major QT of salt tolerance was detected on chromosome 3 (Hamwieh et al., 2011). A total of 21 QTLs were identified, including 4 QTLs related to relative germination rate, 8 QTLs related to relative imbibition rate, and 9 QTLs related to relative germination index (Teng et al., 2022). Based on the analysis of 549 soybean materials, 11 ERF genes were upregulated, among which the ERF158^H1^, ERF166^H2^, and ERF170^H1^ haplotypes were excellent allelic variants, which significantly promoted soybean salt tolerance(Gao et al., 2023). In this study, 257 soybean cultivars with 135 SSR markers were used to perform epistatic association mapping for salt tolerance.A total of 83 QTL-by-environment (QE) interactions for salt tolerance index were detected (Zhang et al., 2014). In the study, a population of 184 recombinant inbred lines (RILs) was utilized to map quantitative trait loci (QTLs) related to salt tolerance. A major QTL related to salt tolerance at the soybean germination stage named qST-8 was closely linked with the marker Sat_162 and detected on chromosome 8 (Yu et al., 2019).

Genome-wide association studies (GWAS) have proven to be a potent tool for investigating complex quantitative traits (Huang et al., 2012). The rapid advancements in modern molecular biology technology have further bolstered the application of molecular marker techniques in various crop breeding domains, including wheat (Kun Dziayi Turhan et al., 2021), rice (He et al., 2023), maize (Ma et al., 2023), sorghum (Gao et al., 2023), rapeseed (Xiao et al., 2023), and other molecular breeding arenas. Soybean exhibits a multitude of complex quantitative traits, often under the control of multiple genes and influenced by both genotype and environmental factors (Kun Dziayi Turhan et al., 2021). Notably, Liang Tengyue et al. (Liang et al., 2023) conducted a genome-wide association analysis on 395 soybean germplasm resources using the GAPIT tool, identifying nine SNPs closely linked to single plant grain weight under low phosphorus conditions. Yang Hao et al. (Yang et al., 2023) conducted a study in the Sichuan-Chongqing region employing 135 SSR markers and 107,081 effective SNP markers for genotyping from 227 soybean varieties and detected 51 and 70 site significantly associated with fertility traits through comprehensive whole-genome association analysis. Compared with the seedling stage, research on the correlation analysis of salt tolerance at the germination stage of soybean has just begun.

The KASP (kompetitive allele-specific PCR) molecular marker is a new SNP typing method based on allele-specific amplification and high-sensitivity fluorescence detection. KASP is characterized by low cost and high throughput, and the accurate double-allele genotyping of SNP and InDel sites through specific matching of primer terminal bases. The method is widely used in molecular marker-assisted selection of rice, wheat, soybean, and other crops (Ertiro et al., 2015; Tian et al., 2018; Cheon et al., 2020; Jiang et al., 2021).

In this study, 283 soybean germplasm resources were used as materials. Under simulated NaCl salt stress, the germination rate, germination energy, and germination index at the germination stage were used as the screening parameters. The integration of genome-wide association analysis (GWAS) allowed us to identify pivotal site associated with soybean salt tolerance in a high-throughput manner. We further harnessed this knowledge to develop KASP markers, leveraging the significant association SNPs to facilitate early selection in the quest for salt-tolerant soybean breeding. This approach significantly alleviates the workload associated with soybean breeding efforts, expediting progress and advancing the field of soybean salt-tolerant molecular breeding. Our findings represent a valuable reference for future research on soybean salt tolerance and the selection and breeding of novel, salt-tolerant soybean varieties.

## Materials and methods

A natural soybean population containingused 283 representative soybean germplasms, including 52 landraces, 212 cultivars, and 19 wild soybeans, was used in this study. To identify an optimal stress concentration for evaluating salt tolerance in soybean germination, eight randomly selected varieties from our study’s test materials underwent a preliminary germination assay. Each variety was subjected to three replicate tests, with a concentration gradient spanning 0, 30, 60, 90, 120, 150, and 180 mM NaCl. Our test results revealed that at a concentration of 150 mmol/L NaCl, the germination rate and other measured parameters exhibited noticeable inhibition. Further increase of the NaCl concentration to 180 mM/L elicited significant disparities in the germination rate, germination energy, and germination index of the materials. Consequently, we identified 180 mmol/L NaCl as the optimal stress concentration for our subsequent experiments.

The germination test was carried out in a dedicated germination room, using a 3×4 grid layout of small squares, with each grid covered by 25 g vermiculite. 50 healthy, full, and pest-free seeds of the same size were used for each condition. 90 ml 0mM and 180 mM Nacl solutions were used for the stress treatment. The seeds were spread on the vermiculite and watered with the treatment solution then covered with 3-4 layers of filter paper soaked with treatment solution. The count of germination seeds was recorded every 24 hours for 7-8 days. This protocol was conducted with three biological replicates per material. Utilizing an established formula, the relative salt damage rate for each germination parameter, including germination rate, germination energy, and germination index, was calculated.

Several key parameters were calculated to assess soybean germination under salt stress conditions, providing insights into salt tolerance:

Germination Rate (GR): The germination rate, expressed as a percentage, was calculated using the formula: GR (%) = (Nt/N) × 100, where Nt represents the number of seeds germinated per grid on day t, and N represents the total number of seeds per grid for testing (unit: %) (Wang et al., 2019).

Germination Index (GI): The germination index was determined using the formula: GI = ∑Gt/Dt, where Gt represents the number of seeds germinated per grid on day t, and Dt signifies the number of days in the germination test up to day (Wang et al., 2019).

Germination Energy (GE): Germination energy, also expressed as a percentage, was calculated as GE (%) = N3/N × 100, where N3 represents the number of seeds germinated per grid on the 3rd day, and N represents the total number of seeds per grid for testing (unit: %) (Wang et al., 2019).

Relative Salt Damage Index (ST): The relative salt damage index was derived using the formula: ST = S/C. Here, C represents the control germination rate, germination index, and germination energy, while S signifies the germination rate, germination index, and germination energy under salt treatment (Wang et al., 2019).

Generalized heritability $h^{2}=\sigma_{g}^{2}/\left(\sigma_{g}^{2}+\frac{\sigma_{ge}^{2}}{n}+\sigma^{2}/nr\right)$ , where $\sigma_{g}^{2}\;(g=1,2,3\ldots 264)$ is the genotype variance of the test material, $\sigma_{ge}^{2}\;\left(e=1,2\right)$ is the variance of the interaction between the genotype and the environment of the test material, $\sigma^{2}$ is the error variance, n is the number of environments, and r is the number of replicates(Yuan et al., 2023).

### Genome-wide association analysis

We resequenced 283 materials in the early stage, achieving an average sequencing depth of 12.4 ×, and yielding a high-density physical map containing a total of 2,597,425 SNPs (Zhang et al., 2021). Genome-wide association analysis was calculated using the GAPIT algorithm package in R (Lipka et al., 2012), and the general linear model (GLM) (Liu et al., 2008) was used for genome-wide association analysis SNPs with -LogP values ≥ 5 are considered to be significant association sites.

### Haplotype and candidate gene analysis

We delineated chromosome intervals based on the target genes, thereby generating a dedicated SNP annotation file and corresponding genotype data. The target interval was methodically classified into five distinct categories, namely the gene-related region (including exons, stopgain, stoploss, splicing, etc.), intronic and UTR regions, upstream and downstream flanking sequences, and intergenic regions. Haplotype analysis was subsequently conducted on these different types of SNP sites. To construct haplotype networks, we employed PopARTv1.7 software. The entire haplotype analysis process was carried out using the R programming language.

After the SNPs significantly associated with salt tolerance traits in soybean germination were identified, we referenced soybean genome information in the online database Phytozome13 (https://phytozome-next.jgi.doe.gov/info/Gmax_Wm82_a2_v1). The genes related to the control of soybean plant height within 120 kb of the SNPs were identified For the analysis of specific population structure, see the research report of our laboratory (Zhang et al., 2021), and the candidate genes were identified by Blastp comparison with the gene sequences in the Arabidopsis genome database.

### Development of KASP markers

Using the Primer-BLAST function of NCBI (https://www.ncbi.nlm.nih.gov/), KASP-PCR amplification primers were designed based on the SNP site S05_41921861 (A/C) and S02_6088007 (A/G) significantly associated with the germination rate, germination energy, germination index of soybean. Each pair of primers consists of two specific forward primers F1 and F2 and a generic reverse primer R. F1 and F2 contain 6-carboxyfluorescein (FAM) and hexachloro-6-methylfluorescein (HEX) fluorescent linker sequences (underlined), respectively. The primer sequences were synthesized by Qingke Biotech (Nanjing).

### PCR procedure used for genotyping with KASP markers

Amplification PCR was performed using KASP V4.0 2×Mastermix (LGC, England), The program is: predenaturation at 94 °C for 15 minutes; Denaturation at 94 °C for 20 s, extension at 61-55 °C for 1 minute, with a decrease of 0.6 °C per cycle for 10 cycles; Denaturation at 94 °C for 20 s, extension at 55 °C for 1 minute, 26 cycles.

### RNA extraction and reverse transcription

Take 0.1g soybean seedlings were quickly ground into powder in liquid nitrogen, and RNA was extracted by Trizol method, Using RNA as template, cDNA was synthesized by HiScript II 1st Strand cDNA Synthesis Kit kit (Vazyme Biotech).

### Determination of candidate gene expression levels

Two salt-tolerant materials were selected to detect gene expression levels, we employed quantitative reverse transcription PCR (RT-PCR) reactions using the Lis system. The internal reference gene selected for this analysis was Tubulin (GenBank: KRG91143.1). The primer sequences for both the candidate gene and the internal reference gene can be found in **Table 1**.

**Table 1:** Sequences of specific primers for qRT-PCR.

serial number	Gene ID	F	R
1	*Glyma.02G067600*	ACAGCATGGGGAGGAAGGTA	CGGAGGAGTGTCCGGATAGA
2	*Glyma.02G067700*	GCGAGTTTGTCCGAGACCAT	TAGCCGTCCCTCCATCGAAT
3	*Glyma.02G068300*	CGATGCACCCAATGATGCTG	TAGGTGGTGGAGACGACGAT
4	*Glyma.02G068700*	TCACAAGGTCGGAAAGCGAG	GTACTGCAACTGCACAAGGC
5	*Glyma.02G068900*	ATGTGCCTACTTGGGCCTTT	CCCGGTTCTGTTTCCCAAGA
6	*Glyma.02G069400*	CCTTGCTGAGCTGCTTTTGG	CTCCTCTTCCAGCTTCCGAC
7	*Glyma.02G070000*	CCAACCTCTTGGATGCCACA	TCCATGTTTGAAAGGTGGCG
8	*Glyma.05G244600*	AGAGAGCGAGTTTGTGCTCC	GCTGGCACTCTTCAACAAGC
9	*Glyma.05G245000*	TGGCTGGTGATCATTGGACC	ATTGATCGTGGCAACGGGAT
10	*Glyma.05G246400*	TGCGTCGTTAAGATGGGCAA	CCCACTGGGGAGGTCTTCTA
11	*Glyma.09G044300*	TGAAAGCGAGCAAGCGAAAC	TGCACTCCTTCAAGGCCAAA
12	*Glyma.09G045200*	CAAGAGCAGCAACAACTCGC	CATTCACCTGGCCCACAAGA

For qRT-PCR, a Gentier96E fluorescence quantitative PCR instrument (purchased from Xi’an Tianlong Co., LTD.) was used for the amplification reaction. The reaction system was prepared as follows: 25 μL 2×Phanta Max Buffer, 1 μL dNTP Mix (10mM each), 3 μL cDNA, 2 μL forward primer, 2 μL reverse primer, 1 μL Phanta Max Super-Fidelity DNA Polymerase (1U/μL), 16 μL water. The amplification detection was conducted in 96-well plates. The reaction procedure was as follows: denaturation at 95°C 10 s; denaturation at 56°C for 20 s, annealing at 72°C for 20 s for a total of 40 cycles.

The expression levels of the target gene were assessed by comparing CT (Cycle threshold) values. When the primers for the target gene of interest exhibited similar amplification coefficients to those of the internal reference gene, the relative expression of the target gene in each sample was calculated using the formula 2^–ΔΔCT^. Here, ΔΔCT was determined as (C_T_, _Target_ - C_T_, _Tubulin_)_genotype_ - (C_T_, _Target_ - C_T_, _Tubulin_)calibrator, allowing for precise evaluation of gene expression levels.

## Results

### Statistical analysis of soybean germination phenotype

This study encompassed the investigation of 283 soybean materials with a focus on salt tolerance traits during the germination period. Three key germination-related traits, namely Germination Rate (GR), Germination Energy (GE), and Germination Index (GI) were recorded. Statistical analysis was conducted on these fundamental indexes of the materials, leading to the derivation of relative indexes, specifically Relative Germination Rate (RGR), Relative Germination energy (RGE), and Relative Germination Index (RGI). The findings are detailed in **Table 2**. Our analysis revealed substantial phenotypic variation in the relative germination traits among the 283 soybean materials, in both 2022 and 2023. Over these two years, RGR, RGE, and RGI exhibited ranges of 0.05 to 1.00, 0.00 to 1.00, and 0.04 to 1.00, respectively. The coefficient of variation (CV) for these traits ranged from 31.81% to 50.60%, and the generalized heritability (h^2^) ranged from 63.87% to 86.48%, highlighting the impact of interactions between plants, environmental factors, and the interplay between plants and the environment on these traits. The observed generalized heritability for each phenotype underscores the quantitative nature of these traits, which are governed by multiple genes. Additionally, it signifies genuine genetic differences in the reproductive period within the population, making them conducive for further analysis, particularly in the context of association studies.

**Table 2:** Descriptive statistics of three germination-related traits in soybean populations under NaCl conditions.

Year	Trait	Max	Min	Mean	SD	CV(%)	h^2^(%)
2022	RGR	1.00	0.05	0.63	0.22	34.90	84.78
	RGE	1.00	0.02	0.45	0.23	50.60	
	RGI	1.00	0.04	0.44	0.18	40.17	63.87
2023	RGR	1.00	0.09	0.64	0.20	31.81	
	RGE	1.00	0.00	0.47	0.24	49.95	86.48
	RGI	1.00	0.04	0.48	0.20	45.60	

84.78, 63.87, 86.48 are the respective value of RGR, RGE and RGI in 2022 and 2023 correspond to generalized heritability.

### Box line plot and frequency distribution analysis of salt tolerance traits during the germination stage

**Figure 1:**
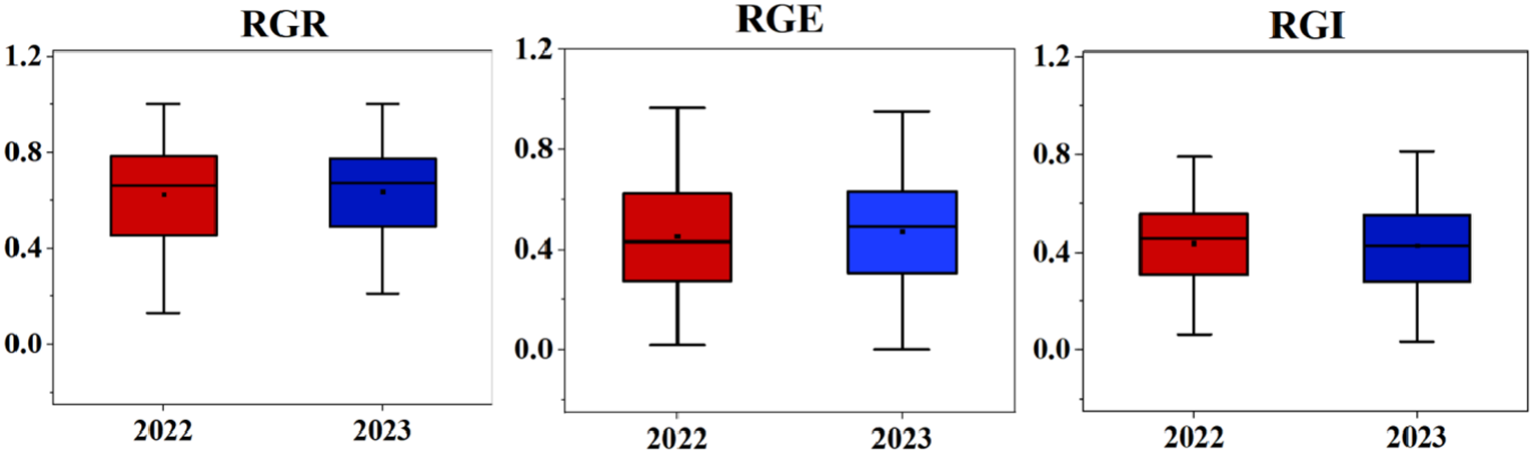
Differences in RGR, RGE, and RGI of soybean germplasm two years. Asterisks indicat significant differences compared with corresponding control(*P < 0.05, **P < 0.01,***P < 0.001).

**Figure 2:**
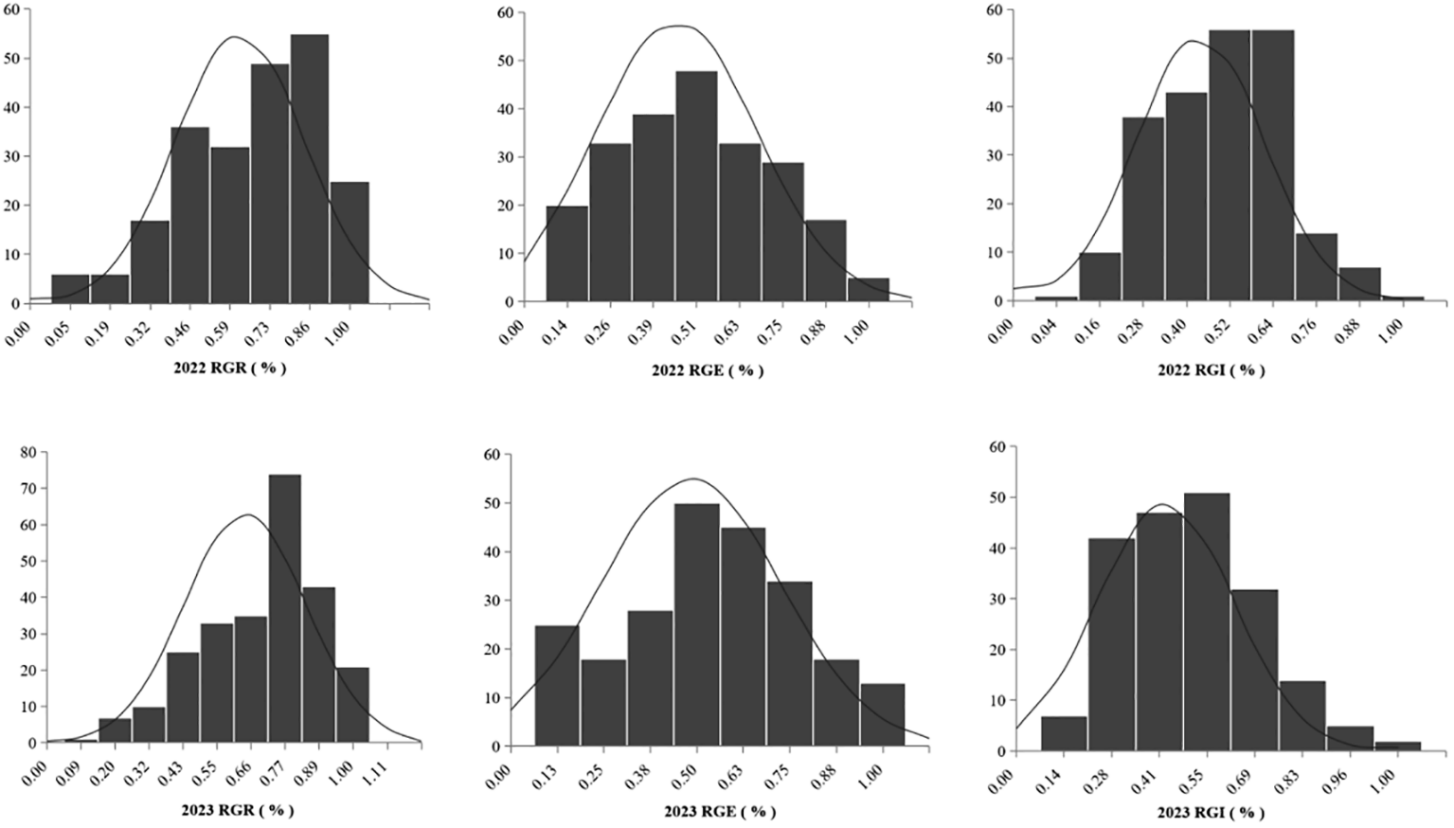
Frequency distribution of RGR, RGE, and RGI in soybean germination.

The box line plot for the relative germination rate, relative germination energy, and relative germination index under two years of salt stress treatment (as shown in **Figure 1**) revealed no disparities in the relative amplitudes for each index between the two years. These calculations for the 283 soybean germplasms were executed using Microsoft Excel 2016. Furthermore, we plotted frequency distribution and density curves (illustrated in **Figure 2**) for the relative germination rate, relative germination energy, and relative germination index. Evident from these representations is the varying extent of suppression in several indicators under salt stress. The histograms displaying the phenotypic data exhibit characteristics resembling approximately normal distribution. This implies that the natural population of soybeans within our study material has rich genetic variation, making it well-suited for subsequent genome-wide association analyses.

### GWAS analysis of salt-tolerance-related traits in soybean germination

**Figure 3:**
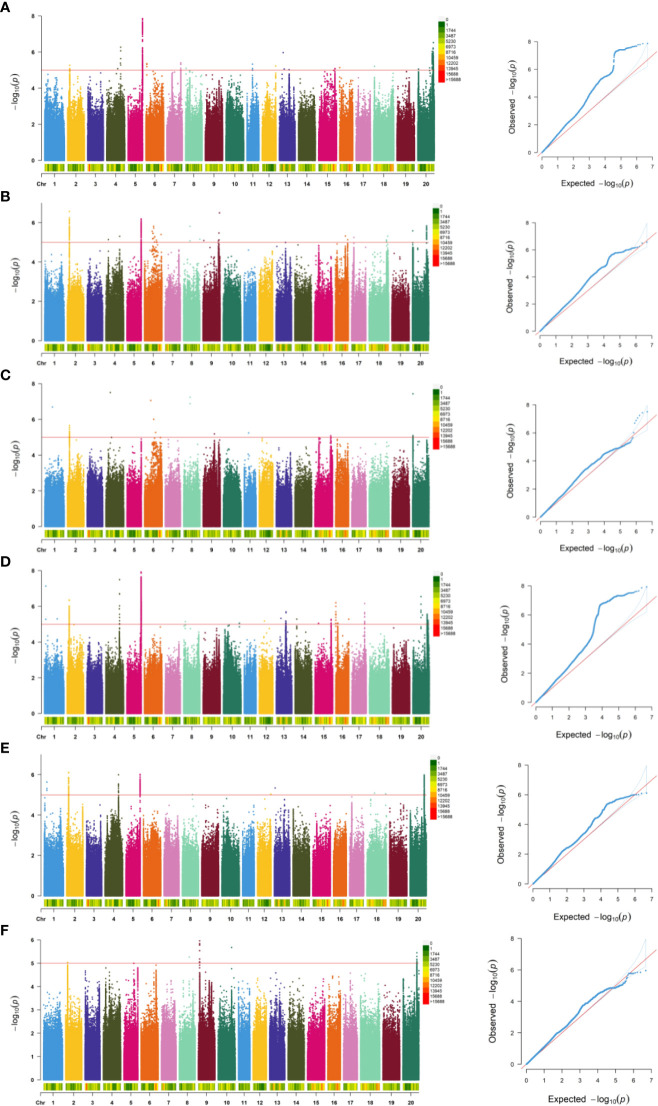
Genome-wide association analysis results of RGR, RGE, and RGI in the natural population of soybean over two years. The solid red lines in the Manhattan plots represent the significant threshold -log10(P)=5.0. **(A-F)** respectively is 2022RGR, 2022RGE, 2022RGI, 2023RGR, 2023RGE, 2023RGI.

Within this study, we integrated the phenotypic results encompassing germination rate, germination energy, germination index, and their corresponding relative indexes (RGR, RGE, RGI) with sequencing data. We used a Generalized Linear Model (GLM) (Liu et al., 2008) to conduct genome-wide association analysis through the GAPIT package in R and created Manhattan plots and QQ plots representing the associated indicators (**Figure 3**). In 2022, a total of 447 SNPs (−log10P>5) closely associated with the soybean germination stage were detected, of which 269 SNP sites were associated with relative germination energy and mainly distributed on chromosomes 2, 5, and 20. Conversely, the sites least associated with the relative germination index were mainly distributed on chromosomes 2, 6, and 20. In 2023, a total of 1841 SNPs closely associated with soybean germination were detected. SNP site was most associated with relative germination rate, with 1512, and mostly distributed on chromosome 5. The sites associated with the relative germination index were mainly distributed on chromosomes 9 and 20. SNPs with significant correlation among traits during germination are detailed in **Table 3**.

**Table 3:** Statistics of GWAS analysis results of germination correlation traits.

Year	Trait	Chromosome	Position interval	Position interval	Peak SNP position	(-log Pmax)	PVE (%)
2022	RGR	4	6	40079022-40641706	40641706	6.27	12.42
		5	137	41755110-42233137	41921861	7.87	16.9
		20	1	2161697-45389200	45231603	6.53	10.67
	RGE	2	82	5966798-6088986	6088007	6.57	11.53
		5	143	41782306-41954581	41912280	6.19	10.76
		20	44	981655-41811347	41792703	5.84	10.06
	RGI	2	27	6062303-6088007	6073517	5.66	9.95
		6	3	18099861-32344653	26921222	7.06	10.66
		20	4	981655-989432	981655	7.43	13.68
2023	RGR	2	116	5966798-6257205	6086046	6.36	10.79
		5	1335	41763734-42233476	41921861	7.92	13.94
		20	61	23510283-44725190	23510343	6.55	10.32
	RGE	2	84	5966798-6257205	6086076	6.11	10.63
		5	133	41871375-41954581	41937985	6.01	10.43
		20	87	41496276-41927972	41792703	5.99	10.37
	RGI	9	13	3852644-3908645	3907313	5.96	12.14
		20	12	41656423-41927972	41759271	5.3	10.92

### Haplotype and candidate gene analysis

**Figure 4:**
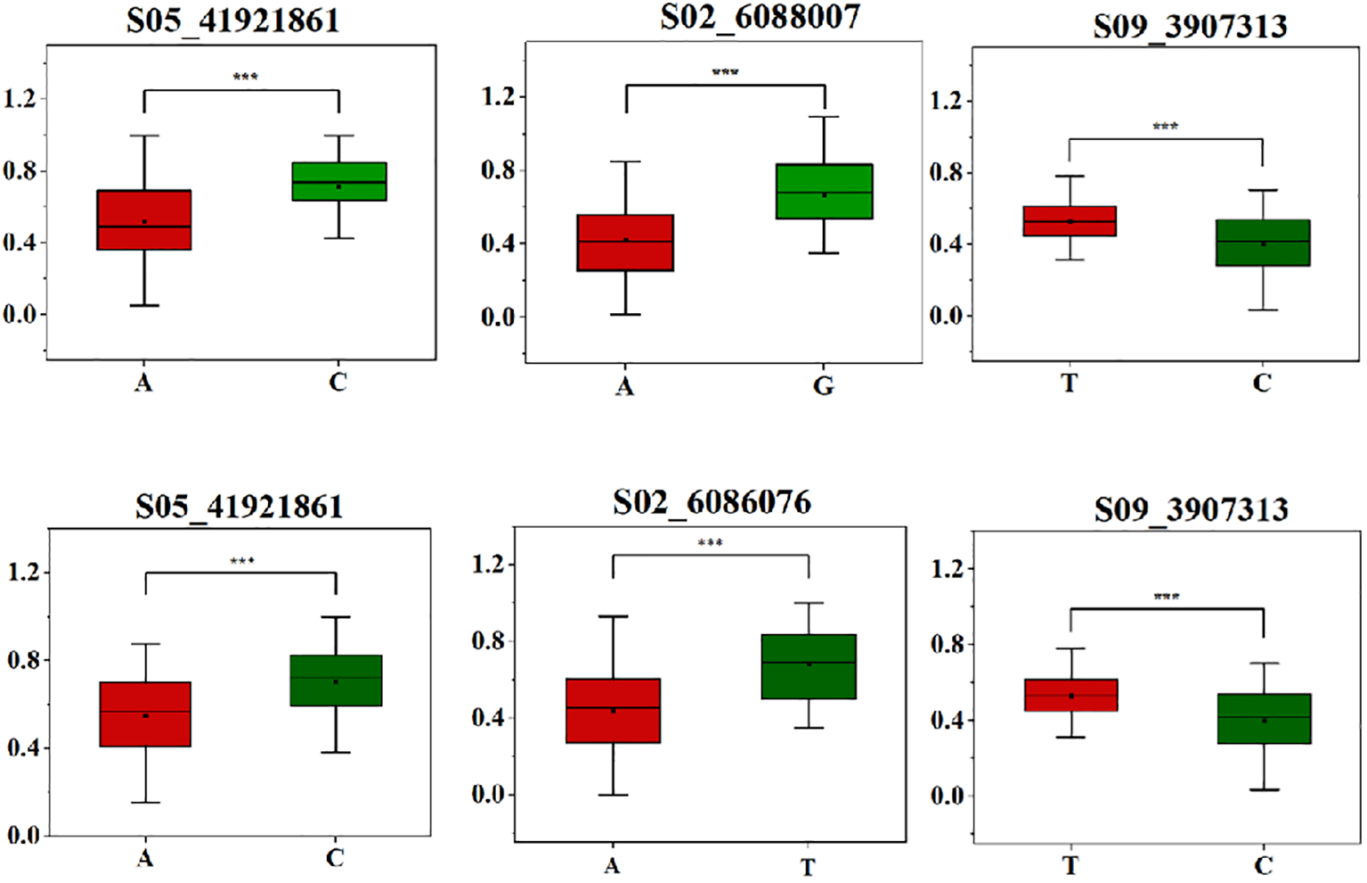
SNP haplotype analysis associated with salt-tolerant traits in a soybean natural population. A is a significant haplotype in 2022, B is a significant haplotype in 2023.Asterisks indicat significant differences compared with corresponding control X axis is the different haplotypes of each point, and Y axis is RGR, RGE and RGI respectively (*P< 0.05, **P< 0.01, ***P< 0.001).

To study the phenotypic impact of allele variations at the most significantly associated SNP sites, haplotype analysis was conducted for these high-threshold SNP sites associated with salt tolerance traits during the germination stage in both 2022 and 2023. It was observed that at the SNP site S05_41921861, the allele variation was A/C, with the average relative germination rate for S05_41921861-A measuring 0.52, a significant reduction compared to 0.71 for S05_41921861-C. Meanwhile, at SNP site S02_6088007, allele variation was A/G, and the average relative germination energy for S02_6088007-A was notably lower at 0.42, as opposed to 0.66 for S02_6088007-G. Lastly, for SNP site S09_3907313, the allele variation was T/C, and the average relative germination index for S09_3907313-C was 0.40, again demonstrating a decrease compared to S09_3907313-T, which exhibited an average of 0.53. This trend in allele variation and its phenotypic effects remained consistent between the years 2022 and 2023 (**Figure 4**).

Candidate gene screening and function prediction were performed in the range of 120 kb upstream and downstream of SNP sites significantly associated with soybean germination tolerance (-log10(P)≥5). Concerning the gene function annotation information of the soybean genome, 12 candidate genes significantly associated with salt tolerance in soybean germination were identified (**Table 4**). These candidate genes were found to be involved in a wide range of critical functions, including coordinating cellular responses, regulating osmotic stress, mitigating oxidative stress, facilitating the clearance of reactive oxygen species (ROS), and functioning as heavy metal ion transporters. Collectively, these genes play pivotal roles in promoting plant development, enhancing stress tolerance, and ensuring normal growth and development of plants.

**Table 4:** Functional annotations of candidate genes related to salt tolerance in soybean germination.

Gene ID	Homologs	Functional annotation
*Glyma.02G067600*	*AT5G13910.1*	Integrase-type DNA-binding superfamily protein
*Glyma.02G067700*	*AT3G02050.1*	K^+^ uptake transporter 3
*Glyma.02G068300*	*AT3G02065.3*	P-loop containing nucleoside triphosphate hydrolases superfamily protein
*Glyma.02G068700*	*AT5G27690.1*	Heavy metal transport/detoxification superfamily protein
*Glyma.02G068900*	*AT5G13870.1*	xyloglucan endotransglucosylase/hydrolase 5
*Glyma.02G069400*	*AT4G18710.2*	Protein kinase superfamily protein
*Glyma.02G070000*	*AT3G04070.2*	NAC domain-containing protein 47
*Glyma.05G244600*	*AT3G18040.1*	MAP kinase 9
*Glyma.05G245000*	*AT1G18180.1*	Protein of unknown function (DUF1295)
*Glyma.05G246400*	*AT3G17980.1*	Calcium-dependent lipid-binding (CaLB domain) family protein
*Glyma.09G044300*	*AT3G16630.2*	P-loop containing nucleoside triphosphate hydrolases superfamily protein
*Glyma.09G045200*	*AT1G56210.1*	Heavy metal transport/detoxification superfamily protein

### Development of KASP markers

**Figure 5:**
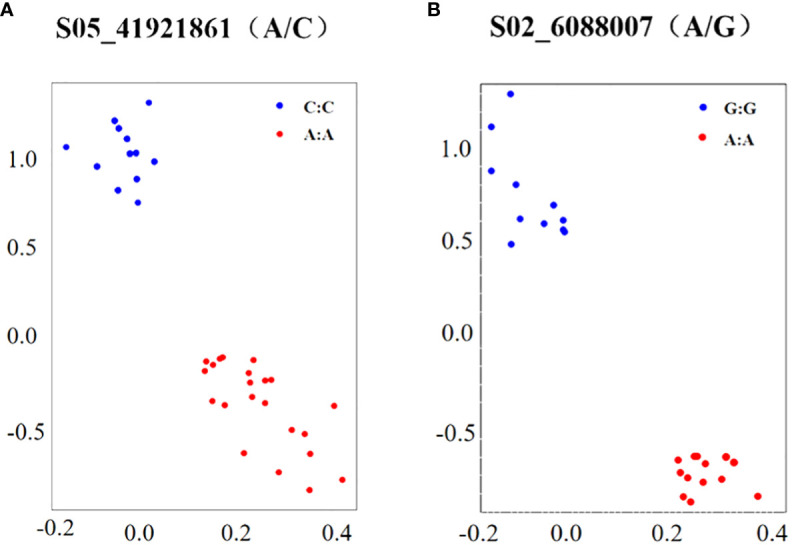
Genotyping of KASP markers. **(A, B)** are genotyping S05_41921861 and S02_6088007, respectively; X-axis and Y-axis scales are the values of the transmitted fluorescence, respectively.

KASP markers were developed for SNP sites S05_41921861 (A/C) and S02_6088007 (A/G), which exhibited significant associations with salt tolerance in soybean germination (**Table 5**). The variation at the S05_41921861 (A/C) site corresponds to the Glyma.05G244600 gene, and its annotation reveals a role in coordinating cellular responses facilitating normal plant growth and development, immune responses, and the capacity to respond to stress. Similarly, the variation at the S02_6088007 (A/G) site is associated with the gene Glyma.02G067600, and its annotation indicates involvement in the upregulation of stress responses. Under salt stress conditions, it triggers the expression the expression of GAOX20, encoding adC7-GA inhibitors. The designed KASP marking series is shown in **Table 5**. The genomic DNA from the selected soybean germplasm was extracted, and the KASP primers, designed for the aforementioned SNP sites, were employed in PCR reactions utilizing genomic DNA as the reaction template. After the completion of the reaction, fluorescence data results were directly read on the real-time PCR system. The genotyping of 48 selected soybean germplasms was executed using the KASP-labeled primers designed for the S05_41921861 (A/C) and S02_6088007 (A/G) sites. The results, illustrated in **Figure 5**, demonstrate the separation of the two different genotypes by PCR.

**Table 5:** Specific primers for KASP.

Primer name	Primer sequences
S05_41921861 F1	**GAAGGTGACCAAGTTCATGCT** GTATAAAGTTGAGGACTG **C**
F2	**GAAGGTCGGAGTCAACGGATT** GTATAAAGTTGAGGACTG **A**
R	TGGTGCTGACTTAGGCACTG
S02_6088007 F1	**GAAGGTGACCAAGTTCATGCT** TATTAATTTATTATTTTTT **G**
F2	**GAAGGTCGGAGTCAACGGATT** TATTAATTTATTATTTTTT **A**
R	TAGCAATGGCATGCACCTCA

The bold text stands for Fluorescent junction sequence.

### Determination of candidate gene expression levels

**Figure 6:**
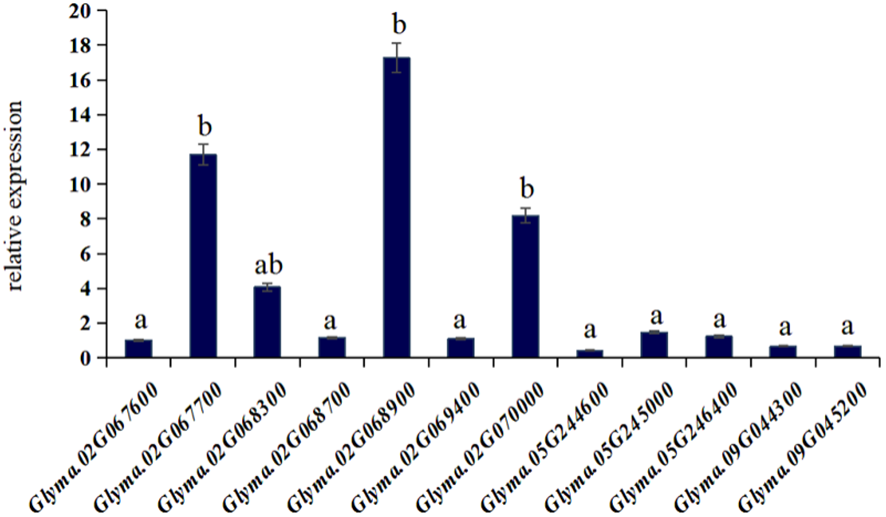
The expression levels of candidate genes. a, b represent significant differences.

The reverse transcription PCR (**Figure 6**) and the rich soybean genome information enabled us to identify *Glyma.02G067700*, Glyma.02G068900, and *Glyma.02G070000* as the genes associated with salt tolerance in soybeans within this population. Incorporating comparative genomics studies of these three candidate genes with other crops and model plants, we uncovered the following insights:

*Glyma.02G067700* codes for a MYB family protein, indicating its role as a key factor in regulatory networks governing development, metabolism, and responses to biotic and abiotic stresses (Shao et al., 2020).

*Glyma.02G068900* encodes xyloglucan endo-transglycosylase/hydrolase (Ph XET/H), which regulates seed germination by facilitating the accumulation of Ph XET protein via GA-mediated pathways. This gene plays a pivotal role in endosperm weakening and embryonic expansion during seed germination, falling within the glycosyl hydrolase family 16 (Jacqueline et al., 1993).

*Glyma.02G070000* codes for an NAC transcription factor, a plant-specific family of transcription factors known for their essential roles in various biological processes (Yuan et al., 2019).

These three genes play an important role in responding to biotic or abiotic stresses, as well as in regulating plant growth and development, and osmoregulation.

## Discussion and conclusion

The research and development of salt-tolerant soybeans for saline-alkali soybean production and the expansion of planting areas through various strategies are pivotal steps in addressing the issue of insufficient soybean production capacity in China. These efforts bear significance for China’s food security. Several studies have contributed to our understanding of salt tolerance mechanisms during soybean germination, shedding light on the physiological changes that occur under salt stress. Hao Xuefeng et al. examined salt tolerance and the salt tolerance mechanism in soybean seeds during germination and found changes in parameters such as SOD, POD, and MDA in the radicle germ in response to increasing NaCl concentrations. This research underscores the existence of specific salt tolerance mechanisms and associated physiological changes during germination (Hao et al., 2013).

Some studies have identified the impact of salt stress on organelle formation, including chloroplasts and endoplasmic reticulum, resulting in varying degrees of influence on growth traits such as root length, hypocotyl length, and lateral root numbers. Ultimately, this process inhibited soybean germination (Liao et al., 2013). demonstrated that high-concentration NaCl stress significantly impeded water absorption in soybean seeds, leading to reduced amylase and protease activity, further elucidating the complexities of salt stress on germination (Xu et al., 2017). Kan found that 22 SSR markers and 11 related QTL sites were closely linked to salt tolerance in soybean germination, and localized on chromosomes 2, 7, 8, 10, 17, and 18 (Kan et al., 2016). research identified a total of 31 salt-tolerant-related QTLs through linkage analysis of ST-IR, ST-GI, ST-GE and ST-GR during the germination stage of an NJIKY population, mainly distributed on chromosomes 1, 2, 7, 8, 10, 15, 17 and 18 (Zhang et al., 2018). Furthermore, Kan used a natural population of 191 local soybean varieties and 1356 SNP markers to perform genome-wide association analysis. Their work identified five candidate genes closely linked to salt tolerance during soybean germination (Kan et al., 2015).

In our pursuit of identifying outstanding salt-tolerant soybean varieties and enhancing soybean yield, this study conducted a comprehensive analysis of germination traits, including germination rate, germination energy, germination index, and their relative values, across a diverse set of 283 soybean germplasm resources subjected to salt stress at the germination stage. Our investigation revealed that the germination traits within this population exhibited a rich and continuous distribution. At the same time, using the high-density SNP physical map combined with phenotype and genotype data for genome-wide association analysis, a total of 1841 SNP sites significantly associated with soybean germination stage were detected on chromosomes 2, 5, 6, 9, and 20. Notably, the loci located on chromosome 5 were repeatedly detected in 2 environments, and the genetic variation explainable by the GWAS signal reached 14.00%, marking it as a prominent genetic locus. MAP kinase 9 may be an effector gene for this site.In the same chromosomal interval as the results of other researchers, there may be allelic variation of the same QTL.Chromosomes 2 and 20 have also been confirmed by previous studies (Zhang, 2014), and the sites associated with chromosome 6, 9 are two new research intervals, which are of great significance for future studies, The related genes of this site should be explored and studied.

Furthermore, sequence comparison of genes within the remaining three significant correlation sites allowed us to predict 12 candidate genes closely linked to the regulation of salt tolerance during the germination stage of soybeans. These candidate genes play roles in the coordination of cellular responses, the regulation of osmotic stress, the attenuation of oxidative stress, the clearance of reactive oxygen species (ROS), and the management of heavy metal ion transport. Collectively, these genes are vital components in plant development, stress tolerance, and the maintenance of normal growth, immune response, and tolerance to abiotic and biotic stresses.

Our findings contribute valuable genetic resources and a solid theoretical foundation for the breeding of salt-tolerant soybeans. They represent a critical step towards addressing the challenges of saline-alkali soybean production and increasing soybean yield, thereby bolstering food security.

## Data availability statement

The original contributions presented in the study are included in the article/supplementary material, further inquiries can be directed to the corresponding author.

## Author contributions

WJ: Writing – original draft, Writing – review & editing. MZ: Writing – review & editing. HZ: Writing – review & editing. XL: Writing – review & editing. WZ: Writing – review & editing. QW: Writing – review & editing. JQ: Writing – review & editing. DX: Writing – review & editing. HC: Writing – review & editing. CS: Writing – review & editing.
